# The Steroidogenesis Inhibitor Finasteride Reduces the Response to Both Stressful and Rewarding Stimuli

**DOI:** 10.3390/biom9110749

**Published:** 2019-11-19

**Authors:** Sean C. Godar, Roberto Cadeddu, Gabriele Floris, Laura J. Mosher, Zhen Mi, David P. Jarmolowicz, Simona Scheggi, Alicia A. Walf, Carolyn J. Koonce, Cheryl A. Frye, Nancy A. Muma, Marco Bortolato

**Affiliations:** 1 Department of Pharmacology and Toxicology, College of Pharmacy, University of Utah, Salt Lake, UT 84112, USA; sean.godar@utah.edu (S.C.G.); roberto.cadeddu@utah.edu (R.C.); gabriele.floris@utah.edu (G.F.); laura.mosher@utah.edu (L.J.M.); simona.scheggi@utah.edu (S.S.); 2 Department of Pharmacology and Toxicology, School of Pharmacy; Lawrence, KS 66045, USA; mizhen@ku.edu (Z.M.); nmuma@ku.edu (N.A.M.); 3 Department of Applied Behavioral Science; University of Kansas, Lawrence, KS 66045, USA; dpj@ku.edu; 4 Cofrin Logan Center for Addiction Research and Treatment; University of Kansas, Lawrence, KS 66045, USA; 5 Department of Cognitive Science, Rensselaer Polytechnic Institute, Troy, NY 12180, USA; walfa@rpi.edu; 6 Department of Psychology; The University at Albany-SUNY, Albany, NY 12222, USA; kooncecj@gmail.com (C.J.K.); cafrye@albany.edu (C.A.F.); 7 Department of Biological Sciences; The University at Albany-SUNY, Albany, NY 12222, USA; 8 Center for Neuroscience, The University at Albany-SUNY, Albany, NY 12222, USA; 9 Comprehensive Neuropsychological Services, Albany, NY 12203, USA

**Keywords:** 5α reductase, depression, anxiety, impulsivity, finasteride, HPA axis

## Abstract

Finasteride (FIN) is the prototypical inhibitor of steroid 5α-reductase (5αR), the enzyme that catalyzes the rate-limiting step of the conversion of progesterone and testosterone into their main neuroactive metabolites. FIN is clinically approved for the treatment of benign prostatic hyperplasia and male baldness; while often well-tolerated, FIN has also been shown to cause or exacerbate psychological problems in vulnerable subjects. Evidence on the psychological effects of FIN, however, remains controversial, in view of inconsistent clinical reports. Here, we tested the effects of FIN in a battery of tests aimed at capturing complementary aspects of mood regulation and stress reactivity in rats. FIN reduced exploratory, incentive, prosocial, and risk-taking behavior; furthermore, it decreased stress coping, as revealed by increased immobility in the forced-swim test (FST). This last effect was also observed in female and orchiectomized male rats, suggesting that the mechanism of action of FIN does not primarily reflect changes in gonadal steroids. The effects of FIN on FST responses were associated with a dramatic decrease in corticotropin release hormone (CRH) mRNA and adrenocorticotropic hormone (ACTH) levels. These results suggest that FIN impairs stress reactivity and reduces behavioral activation and impulsive behavior by altering the function of the hypothalamus–pituitary–adrenal (HPA) axis.

## 1. Introduction

Steroid 5α-reductase (5αR) is the enzyme catalyzing the saturation of the 4,5-double bond of the A ring of testosterone, progesterone, and deoxycorticosterone, as well as other ketosteroids [1,2]. The products of this metabolic process, 5α-dihydrotestosterone (DHT), 5α-dihydroprogesterone (DHP), and 5α-dihydrodeoxycorticosterone (DHDOC) are further converted by 3α-hydroxysteroid oxidoreductase into 5α-androstan-3α, 17β-diol (3α-diol), tetrahydroprogesterone (allopregnanolone; AP), and tetrahydrodeoxycorticosterone (THDOC), respectively. These neuroactive steroids act as positive allosteric modulators of GABA_A_ receptors [3,4] and play key roles in the orchestration of reactivity to stress and other environmental stimuli as well as in the pathophysiology of depression and anxiety [5,6,7,8,9]. In addition, 5αR plays a key role in the degradation of glucocorticoids, such as corticosterone and cortisol, into their 5α-reduced metabolites [10].

The prototypical 5αR inhibitor, finasteride (FIN; N-(2-methyl-2-propyl)-3-oxo-4-aza-5α-androst-1-ene-17β carboxamide), was originally approved in the 1990s for the treatment of benign prostatic hyperplasia and male-pattern baldness [2]. These therapeutic actions reflect the best-characterized mechanism of action of FIN, namely the reduction of DHT synthesis in plasma and scalp [11,12]. In addition to this effect, FIN impairs the synthesis of other neuroactive steroids, including AP and 3α-diol [13,14,15]. Although FIN is generally well-tolerated, post-marketing reports of adverse psychological events have led to growing concerns about the safety profile of this drug. Several studies have substantiated that FIN increases the risk for depressive symptoms in a subset of vulnerable patients [16,17,18]. In addition, several patients have reported that these psychological complications can persist even after FIN discontinuation [18,19,20,21,22,23], prompting some authors to coin the term “post-finasteride syndrome” (PFS) to define this condition. Altogether, this emerging evidence has led several national agencies to issue warnings about the potential for depression and other psychological sequelae [22,23].

The pathophysiology of PFS and other psychological complications of FIN remain unclear, in view of selection bias in many published clinical studies and high nocebo effect, as well as suboptimal safety information [22,23]. A full characterization of the behavioral effects of FIN is particularly important, given that preliminary reports have indicated that FIN may also have therapeutic properties for several neuropsychiatric conditions characterized by poor impulse control and excessive externalizing manifestations, including Tourette’s syndrome and pathological gambling [2,24,25]. Based on this background, the present study tested the behavioral effects of FIN in a broad battery of standardized behavioral tests in rats, in order to assess its effects on complementary aspects of mood regulation, anxiety, impulse control, and stress reactivity. The latter was further characterized by measuring plasma adrenocorticotropin (ACTH) levels and corticotropin-releasing hormone (CRH) mRNA in the paraventricular nucleus (PVN) of the hypothalamus.

## 2. Materials and Methods

### 2.1. Animals

The experiments in this study were performed using Long–Evans rats (Charles River Laboratories, Raleigh, NC, USA) weighing 250–350 g and housed in groups of 3–4 per cage. Unless stated otherwise for specific experimental manipulations, rats were kept with ad libitum access to food and water. For all tests, animals were used only once. Experimental manipulations were carried out in the animals’ dark cycle between 10:00 AM and 06:00 PM. All handling and experimental procedures were performed in compliance with the National Institute of Health guidelines and approved by the local Institutional Animal Care and Use Committees. (Protocol 19-05005).

### 2.2. Drugs

FIN was purchased from Carbosynth Limited (Compton, UK) and suspended in a vehicle (VEH) solution containing 5% DMSO, 5% Tween80, and 90% sterile saline (SAL; 0.9% NaCl).

### 2.3. Orchiectomy

Gonadectomy and sham surgeries were performed as previously described [26] under aseptic conditions, using a combination of ketamine (80 mg/kg; Fort Dodge Animal Health, Fort Dodge, IA, USA) and xylazine (10 mg/kg; Bayer Crop. Shawnee Mission, KS, USA) for anesthesia. For both operations, the sac of the scrotum and underlying tunica were incised; orchiectomy was performed by bilateral ligation of the vas deferens and removal of the testes. Incisions were sutured closed.

### 2.4. Locomotor Activity

Locomotion was tested in a square force plate actometer, as previously described [27]. The apparatus consisted of a white load plate (42 × 42 cm) surrounded on all four sides and covered by a clear Plexiglas box (30 cm tall). Four force transducers placed at the corners of each load plate were sampled 100 times/s, yielding a 0.01 s temporal resolution, a 0.2 g force resolution, and a 2 mm spatial resolution. Custom software directed the timing and data-logging processes via a LabMaster interface (Scientific Solutions Inc., Mentor, OH, USA). Total distance traveled was calculated as the sum of the distances between coordinates of the location of center of force, recorded every 0.5 s over the recording session. Animals were placed in the center and their behavior was monitored for 30 min. The test was performed in complete darkness to avoid potential anxiety-related confounds.

### 2.5. Defensive Withdrawal

Defensive withdrawal test was performed with two alternative variants of our previously described protocol [28]. In the first version, adult rats were placed in a small rectangular black Plexiglas chamber (23 cm height × 15 cm width × 25 cm depth) opened at one end, located 20 cm from the wall of a cylindrical, stainless-steel, white, open field (124 cm diameter; 56 cm height), placed under bright light (200 lux), and video-recorded for 15 min. A separate cohort underwent the same procedure, but was initially placed within the open arena, in a position diametrically opposed to the chamber. Behavioral analyses were performed by blinded observers. The latency to leave the chamber and the percentage of time spent in the open field were measured. FIN and its vehicle were administered 45 min before testing.

### 2.6. Novelty-Induced Hypophagia

Rats were single housed and carried within their cage to a dimly illuminated room (50 lux under red light) for four consecutive days. After a 30 min acclimation period, they were presented with a highly palatable food (two cheese puffs made of corn flour, hydrogenated vegetable fat, cheese powder and salt) [29]. Following this training, a group of animals were transferred to a novel cage (20 cm × 29 cm × 35.5 cm) and moved to a brightly lit room (500 lux, under white light). The other rats were maintained in their home cages under habitual light conditions (50 lux, under red light). Animals in either condition were then treated with either FIN or its vehicle; 45 min after treatment, they were given two cheese puffs and their behavior was videotaped. The latency to eat and total consumption were recorded by an observer blinded to treatment.

### 2.7. Social Interaction

Social interaction was tested as previously described [30]. Male rats were placed in a novel round aluminum chamber (diameter: 124 cm; height: 56 cm) 45 min after treatment with FIN or its vehicle. Social interaction was tested against either foreign counterparts or cage mates. Behavior was videotaped, and the duration of sniffing in the genital, mid-sectional, and facial areas, as well as grooming and rearing behavior, were measured by an observer blinded to treatment.

### 2.8. Delay Discounting

Delay discounting procedure was performed with a modification of previously described protocol [31]. Subjects were 18 male rats, maintained at 85% of their ad libitum weights by restricting access to food (Teklad Diet 8064, Harlan Laboratories Inc., Indianapolis, IN, USA). Briefly, sessions occurred in eight identical operant conditioning chambers (Med Associates, St. Albans, VT, USA) fitted with two retractable levers, cue lights, a houselight, and a pellet dispenser delivering grain-based pellets (45 mg; Bio-Serv, Frenchtown, NJ, USA). All experimental events were programmed and recorded using MED-PC IV software. Sessions consisted of 32 trials, divided into four 8 trial blocks. The beginning of a block was signaled by houselight flashing; when each trial began, the houselight was continuously illuminated and either one (forced choice trials) or both (free choice trials) levers were inserted into the chamber. Each block consisted of two forced choice trials (during which the rats pressed one of the levers and received the consequence associated with that lever), followed by six free-choice trials between two alternatives: “Smaller–Sooner” (SS), delivering one pellet at 0 s delay, or “Larger–Later” (LL), delivering three pellets at progressively larger delays. Each trial lasted 60 s, inclusive of the response period, the delay (during which levers were retracted), and the intertrial interval (during which levers were retracted). The position of the SS and LL levers was counterbalanced among subjects. For each session, the four blocks of trials differed by the delay on the LL choice, presented in increasing order of delay during each session. Training lasted 30 days and included the following sessions: (1) all trial blocks with 0 s LL delays; (2) trial blocks with 0 s, 1 s, 2 s, and 4 s LL delays; (3) trial blocks with 0 s, 1 s, 10 s, 20 s LL delays; and (4) trial blocks with 0 s, 10 s, 20 s, 40 s LL delays. Rats progressed from each session to the next upon verification of their statistical stability (i.e., no significant differences across three subsequent sessions) Testing with FIN (25 mg/kg, IP, 45 min before session) or its vehicle was performed under these conditions over 11 sessions.

### 2.9. Wire-Beam Bridge Test

Testing was performed on a methodological variant of our wire-beam bridge protocol [32]. The apparatus consisted of two metal platforms (start and end) raised at 130 cm from the floor, placed 100 cm apart, and connected by a horizontal, aluminum wire-beam bridge. The start platform was limited by a wall to limit movements in any other direction. The bridge (4 cm wide) consisted of two parallel, 0.1 cm thick beams, connected perpendicularly by 40 crossties (placed 2.5 cm apart), and was highly flexible with a downward deflection of 2 cm per 100 g load at the center point. Rats were then placed on the start platform, and the latency to cross as well as the number of bridge crossings were recorded. Cut-off test time to cross the bridge was set at 10 min.

### 2.10. Forced-Swim Test

The forced-swim test was performed as previously described [33]. Briefly, rats were tested in clear Plexiglas cylinders (45.7 × 30 cm in diameter) filled with water to 30 cm. The water temperature was maintained at 25 °C. Testing lasted 10 min. Environmental light was kept at 300 lux. Animals were video recorded, and the duration of immobility (s) and the latency to immobility (s) were measured.

### 2.11. Saccharin Preference

Rats were initially tested for baseline saccharin consumption and preference. They were deprived of food and water for 15 h prior to the test, starting 1 h before the onset of the dark phase. Each animal was given access to one pre-weighed bottle containing a 0.1% saccharin solution in tap water. One hour later, the bottle was removed and weighed again, and food and water were placed back in the cage. Saccharin consumption tests were repeated every 3 to 4 days for the following 2 weeks. After stabilization of saccharin consumption, rats were given two bottles, containing a 0.1% saccharin solution (presented on either the left or the right side of the cage, in counterbalanced order) and water, respectively, for 4 h. Saccharin preference was assessed as the ratio of saccharin solution/total liquid consumed by each rat. After saccharin preference reached stability (assessed by <10% variation over three consecutive sessions), rats were assigned to three groups (matching for preference) to be treated with FIN (25–50 mg/kg, IP) or its vehicle. Saccharin solution and water consumption was assessed every 30 min for 4 h.

### 2.12. Quantification of CRH and ACTH

Plasma ACTH levels and CRH mRNA in the PVN were measured to assess hypothalamus–pituitary–adrenal (HPA) axis function. Thirty min after FST, rats were decapitated, and brains were rapidly harvested and frozen in liquid nitrogen. Trunk blood was collected into tubes containing 0.5 mL of 0.3 M EDTA. Plasma aliquots and brains were stored at −80 °C until use. ACTH levels were determined by radioimmunoassay as previously described [34,35]. Radioactive ^125^I ACTH (specific activity: 2200 Ci/mmol) was obtained from Perkin Elmer (Waltham, MA, USA) and DiaSorin (Stillwater, MN, USA), respectively. The intra-assay coefficient of variation was 2.17 for the ACTH assay. The PVN was punched out from 300 μm thick sections prepared using a cryostat microtome. Total RNA was isolated using the RNeasy Mini Kit (Qiagen Sciences, Valencia, CA, USA) and preserved in RNAlater^®^ solution (Life Technologies, Carlsbad, CA, USA) according to the manufactures’ protocol. First strand cDNA was synthesized using Superscript III Reverse Transcriptase (Life Technologies, Carlsbad, CA, USA). Real-time PCR amplification was performed using 7500 Real-Time PCR System and SYBR green PCR master mix (Life Technologies, Carlsbad, CA, USA). All samples were run in triplicate. The primers were synthesized by Life Technologies (Carlsbad, CA, USA). The forward primer for CRH was CTGATCCGCATGGGTGAAGA and the reverse primer was CAGCAACACGCGGAAAAAGT. The mRNA levels were normalized to TATA-box binding protein (TBP) mRNA. ΔCt was calculated as the CRH mRNA − TBP mRNA for each sample; ΔΔCt was calculated as ΔCt for the experimental condition − ΔCt for the control condition, for CRH.

### 2.13. Statistical Analyses

Normality and homoscedasticity of data were verified by the Kolmogorov–Smirnov test. Parametric and non-parametric statistical analyses of behavioral parameters were performed via one-way ANOVAs or Kruskal–Wallis test. Analyses of delay discounting, saccharin preference, and CRH/ACTH levels were performed via two-way ANOVAs. All *post-hoc* analyses were performed via Tukey’s test. Significance was set at *p* < 0.05.

## 3. Results

### 3.1. FIN Reduced Exploratory and Appetitive Behavior at Doses That Did Not Affect Locomotor Activity

**Figure 1:**
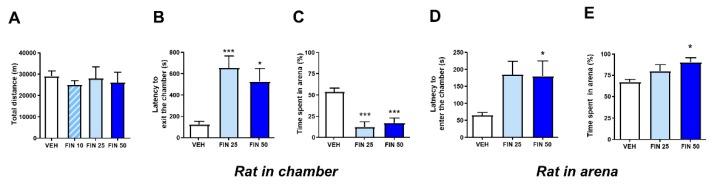
Finasteride (FIN) reduced exploratory drive in the defensive withdrawal paradigm. FIN did not affect locomotor activity in rats tested in an actometer under total darkness; *n* = 6–7/group (**A**). In the defensive withdrawal paradigm, FIN increased the latency to exit from a protected chamber and enter a brightly lit open arena (**B**), as well as the percentage of time spent in the arena itself, *n* = 12/group (**C**). However, when animals were first placed in the open arena, FIN increased the latency to enter the protected chamber (**D**) and prolonged the percentage of time spent in the open arena *n* = 8/group (**E**). * *p* < 0.05, *** *p* < 0.001 in comparison with rats treated with vehicle (VEH). Doses of finasteride are indicated in mg/kg (IP).

The effects of FIN (10, 25, and 50 mg/kg, IP) were first tested on locomotor behavior under complete darkness, to test its effects on intrinsic locomotor activity. FIN did not affect the total distance traveled by rats at any tested dosage (One-way ANOVA, F_3,22_ = 0.18, NS; Figure 1A). To characterize the effect of FIN on anxiety-related indices, rats were first tested in defensive withdrawal, a paradigm that captures the propensity of rats to exit a protected small chamber and enter a brightly lit open arena. FIN (25–50 mg/kg, IP) dose-dependently increased the latency to withdraw from the protected chamber (one-way ANOVA: F_2,35_ = 9.018, *p* < 0.001; *post-hoc* comparisons: VEH vs. FIN 25, *p* < 0.001; VEH vs. FIN 50, *p* < 0.05, Figure 1B) and the percentage of time spent in the open arena (one-way ANOVA: F_2,35_ = 15.71, *p* < 0.001; *post-hoc* comparisons: VEH vs. both FIN 25 and FIN 50: *p’s* < 0.001; Figure 1C).

A reduced proclivity to exit the enclosed chamber may signify either an increased anxiety-like response or a reduced exploratory drive; thus, to tease out the meaning of the observed behavioral effects of FIN, we repeated the same test with a second cohort of rats, which were initially placed in the open arena. In this version of the paradigm, FIN (50 mg/kg, IP) increased the latency to enter the protected chamber (one-way ANOVA: F_2,21_ = 4.35, *p* < 0.05; *post-hoc* comparisons: VEH vs. FIN 50, *p* < 0.05; Figure 1D) and prolonged the percentage of the time spent in the open arena (one-way ANOVA: F_2,21_ = 4.70, *p* < 0.05; *post-hoc* comparisons: VEH vs. FIN 50, *p* < 0.05; Figure 1E). These results suggest that FIN reduced behavioral activation irrespective of the anxiogenic characteristics of the environment.

**Figure 2:**
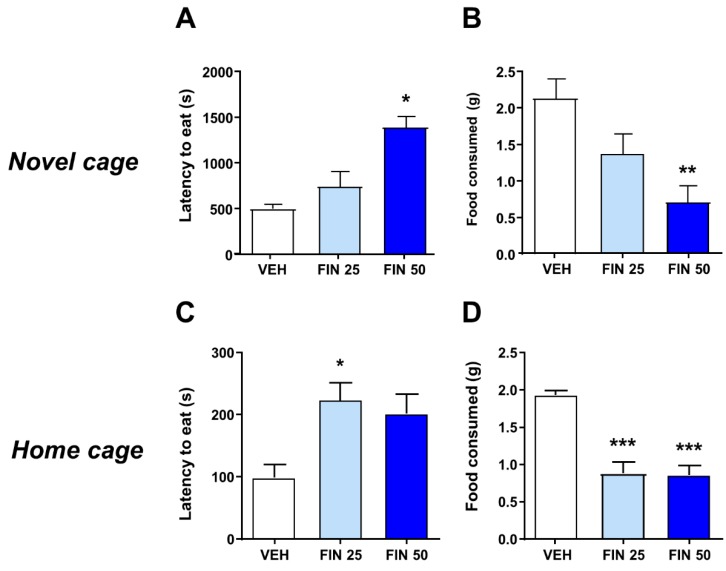
Finasteride (FIN) reduced appetitive motivation in the novelty-induced hypophagia test. In a novel cage, FIN-treated rats displayed an increased latency to consume palatable food (**A**) and reduced amounts of food consumed (**B**); *n* = 10–11/group. Similar outcomes, however, were also observed in home cages; *n* = 9/group (**C,D**). * *p* < 0.05, ** *p* < 0.01, *** *p* < 0.001 in comparison with rats treated with vehicle (VEH). Doses of FIN are indicated in mg/kg (IP).

We then tested the effects of FIN in the novelty-induced hypophagia test. FIN-treated rats displayed an increased latency to consume palatable food in a novel cage (Kruskal–Wallis, H_2_ = 6.65, *p* < 0.05; *post-hoc* comparisons: VEH vs. FIN 50, *p* < 0.05; Figure 2A) and decreased the amount of food consumed (One-way ANOVA; F_2,28_ = 6.98, *p* < 0.01; *post-hoc* comparisons: VEH vs. FIN 50, *p* < 0.01; Figure 2B). To verify whether the greater food avoidance induced by FIN reflected an actual increase in contextual neophobia, rather than a generalized reduction in appetitive drive, we used a separate cohort of rats to test whether FIN also reduced the consumption of the same palatable food in the home cage. Notably, FIN-treated rats exhibited a marked increase in the latency to consume food (one-way ANOVA; F_2,24_ = 3.66, *p* < 0.05; *post-hoc* comparisons: VEH vs. FIN 25, *p* < 0.05; Figure 2C) and reduce the amount of food consumed (one-way ANOVA; F_2,24_ = 13.36, *p* < 0.001; *post-hoc* comparisons: VEH vs. FIN 25 and 50, *p*’s< 0.001; Figure 2D), again supporting the conclusion that FIN reduces incentive motivation towards rewarding stimuli, rather than increasing anxiety.

### 3.2. FIN Reduced Sociability in Rats

**Figure 3:**
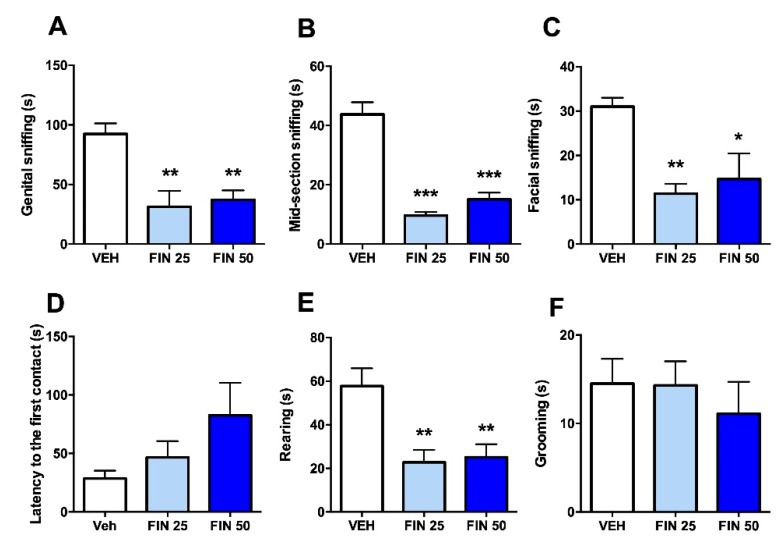
Finasteride (FIN) reduced social interaction with foreign social counterparts. When encountering foreign rats, FIN-treated rats exhibited significant reductions in the duration of genital (**A**), mid-section (**B**), and facial sniffing (**C**), but no changes in the latency to the first social approach, *n* = 9/10/group (**D**). In addition, FIN reduced rearing (**E**), but not grooming responses (**F**). * *p* < 0.05, ** *p* < 0.01, *** *p* < 0.001 in comparison to rats treated with vehicle (VEH). Doses of FIN are indicated in mg/kg (IP).

The effects of FIN on social interaction with foreign rats were examined to verify the impact of this drug on social anxiety. FIN significantly reduced the duration of genital (one-way ANOVA, F_2,25_ = 9.31, *p* < 0.001; *post-hoc* comparisons: VEH vs. FIN 25 and 50, *p* < 0.01; Figure 3A), mid-section (one-way ANOVA, F_2,25_ = 46.39, *p* < 0.001; *post-hoc* comparisons: VEH vs. FIN 25 and 50, *p* < 0.001; Figure 3B), and facial sniffing (one-way ANOVA, F_2,25_ = 6.49, *p* < 0.01; *post-hoc* comparisons: VEH vs. FIN 25, *p* < 0.01; VEH vs. FIN 50, *p* < 0.05; Figure 3C); however, FIN did not affect the latency to the first social approach (one-way ANOVA, F_2,25_ = 1.95, NS; Figure 3D). FIN also reduced rearing (one-way ANOVA, F_2,25_ = 8.28, *p* < 0.01; *post-hoc* comparisons: VEH vs. FIN 25 and 50, *p* < 0.01; Figure 3E), but not grooming behavior (one-way ANOVA, F_2,25_ = 0.38, NS; Figure 3F).

To understand whether the social avoidance observed in FIN-treated rats was related to social anxiety or to a reduction of prosocial drive, we tested the effects of FIN on social interaction with familiar rats (weight-matched cage mates).

**Figure 4:**
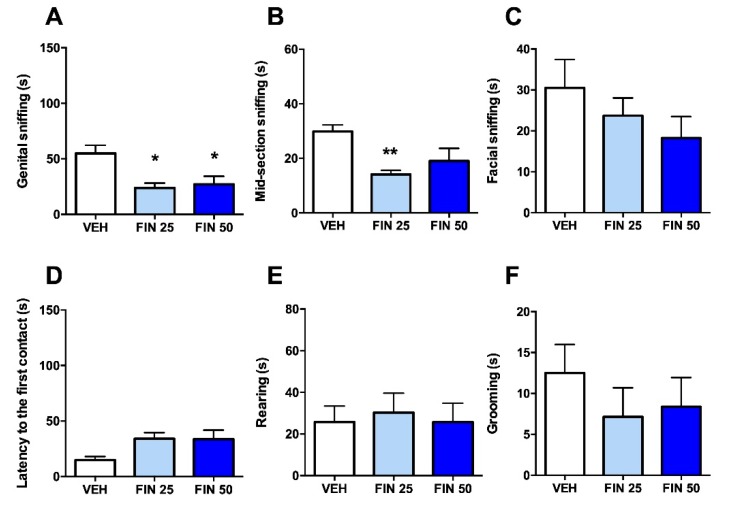
Finasteride (FIN) reduced social interaction with familiar social counterparts. FIN decreased the duration of the exploration of genital area (**A**) and mid-section (**B**), but not facial areas (**C**) of cage mates. Furthermore, FIN had no effect on the latency to the first social approach (**D**), duration of rearing behavior (**E**) and grooming; *n* = 7–8/group (**F**). * *p* < 0.05, ** *p* < 0.01 in comparison with rats treated with vehicle (VEH). Doses of FIN are indicated in mg/kg (IP).

In this version of the test, both doses of FIN elicited a significant reduction of genital sniffing (one-way ANOVA, F_2,20_ = 6.58, *p* < 0.01; *post-hoc* comparisons: VEH vs. FIN 25 and 50, *p* < 0.05, Figure 4A). Furthermore, the 25 mg/kg dose of FIN reduced mid-section sniffing (one-way ANOVA, F_2,20_ = 5.98, *p* < 0.01; *post-hoc* comparisons: VEH vs. FIN 25, *p* < 0.01; Figure 4B). None of the other behavioral parameters, however, were affected by FIN, irrespective of the dose (facial sniffing: one-way ANOVA, F_2,20_ = 1.19, NS; latency to the first contact: one-way ANOVA, F_2,20_ = 3.29, NS; rearing: one-way ANOVA, F_2,20_ = 0.086, NS; grooming: one-way ANOVA, F_2,20_ = 0.627, NS).

### 3.3. FIN Reduced Impulsivity and Risk-Taking Responses

**Figure 5:**
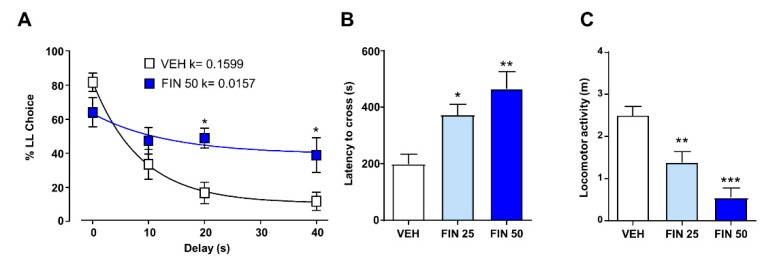
Finasteride (FIN) decreased impulsive and risk-taking responses. In the delay-discounting paradigm (**A**)**,** FIN decreased the discounting rate corresponding to longer delays (20 and 40 s); *n* = 11–12/group. In the wire-beam bridge, FIN-treated rats showed longer latency to cross the bridge (**B**) and an overall reduction in the distance traveled on the apparatus (**C**); *n* = 10/group (C). * *p* < 0.05, ** *p* 0.01, *** *p* < 0.001 in comparison with rats treated with vehicle (VEH). Doses of FIN are indicated in mg/kg (IP).

We next tested whether FIN affected risk taking and other facets of impulsivity. In a delay-discounting paradigm, two-way ANOVA analyses of %LL responses revealed an interaction between FIN and delay (F_3,80_ = 4.24, *p* < 0.05). *Post-hoc* analyses revealed that FIN induced a shift toward SS reward choices in correspondence of longer delays (20 and 40 s) (*p*’s < 0.05; Figure 5A), signifying a reduction in delay discounting. No omissions of lever presses were observed in any experimental group. FIN also reduced risk-taking behavior in the suspended wire-beam bridge paradigm, as signified by a longer latency to cross the bridge (one-way ANOVA, F_2,27_ = 7.90, *p* < 0.01; *p* < 0.05 and *p* < 0.01 for comparisons between 25 mg/kg and 50 mg/kg FIN, respectively: Figure 5B), as well as a reduced distance traveled on the apparatus (one-way ANOVA, F_2,27_ = 17.02, *p* < 0.001; *p* < 0.05 and *p*< 0.01; for comparisons between 25 mg/kg and 50 mg/kg FIN, respectively; Figure 5C).

### 3.4. FIN Reduced Saccharin Preference

**Figure 6:**
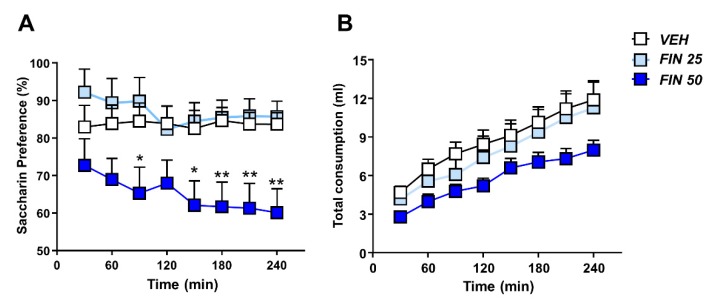
Finasteride reduced saccharin preference. The 50 mg/kg dose of FIN reduced saccharin preference in the saccharin preference test (**A**) without modifying total liquid consumption (**B**); *n* = 10–11/group; * *p* < 0.05, ** *p* < 0.01 in comparison with rats treated with vehicle (VEH). Doses of FIN are indicated in mg/kg (IP).

We then determined whether FIN affected the preference for rewarding stimuli. To this end, the influence of FIN on behavioral reactivity to a sweet solution was assessed by measuring the preference for a saccharin (0.1%) solution. Two-way, repeated-measure ANOVA showed a significant effect of the treatment (F_2,28_ = 5.120, *p* < 0.05) and time (F_7,196_ = 3.24, *p* < 0.01), as well as their interaction (F_14,196_ = 1.73, *p* = 0.05). A deficit in the expression of saccharin preference was observed in rats treated with 50 mg/kg FIN between 180 and 240 min after treatment (*p*’s < 0.05; Figure 6A). Conversely, the 25 mg/kg dose did not significantly affect saccharin preference. No significant effects of FIN were observed in the total liquid consumption (treatment: F_2,28_ = 2.65, NS; time × treatment interaction: F_14,196_ = 0.98, NS; Figure 6B).

### 3.5. FIN Reduced Stress Coping Behavior and Suppressed HPA Axis Responses

**Figure 7:**
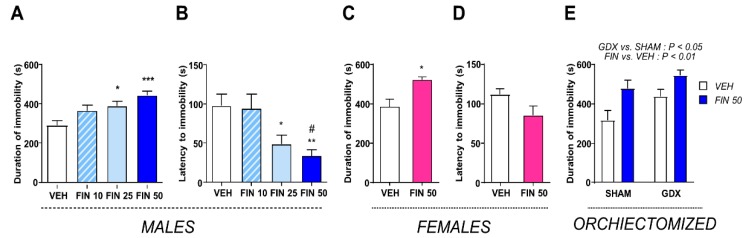
Finasteride (FIN) reduced stress coping behavior in the forced-swim test. In male rats, FIN increased the duration of immobility (**A**) and reduced the latency to immobility (**B**); *n* = 12/group. In female rats, FIN affected only the duration of immobility (**C**), but not the latency (**D**); *n* = 8–9/group. The depressogenic effect was not modified by gonadectomy (**E**); *n* = 9–11/group (GDX) (**E**). * *p* < 0.05, ** *p* 0.01, *** *p* < 0.001 in comparison with rats treated with vehicle (VEH); # *p* < 0.05 in comparison with FIN (10 mg/kg, IP). Doses of FIN are indicated in mg/kg (IP).

Finally, we investigated the effects of FIN on acute stress coping. To this end, the effects of different doses of FIN (10, 25, and 50 mg/kg, IP) were evaluated in the FST. In males, FIN treatment significantly affected both the duration of immobility (one-way ANOVA; F_3,40_ = 6.67, *p* < 0.001; Figure 7A) and the latency to immobility (one-way ANOVA, F_3,40_ = 6.28, *p* < 0.01; Figure 7B). *Post-hoc* analysis revealed that FIN 25 and 50 mg/kg increased total immobility (*p* < 0.05 and *p* < 0.001 compared to VEH, respectively; Figure 7A) and decreased the latency to immobility (*p* < 0.05 and *p* < 0.01 compared to VEH, respectively; Figure 7B). To examine whether the effects of FIN on the FST could be reflective of changes in peripheral levels of testosterone and its derivatives, we then tested the effects of this drug in male orchiectomized and female rats. In females, FIN 50 increased total immobility (Student’s *t*-test, *p* < 0.05; Figure 7C), although it did not significantly affect the latency to immobility (Figure 7D). The analysis of the effects of FIN on castrated males revealed that both orchiectomy (two-way ANOVA: F_1,36_ = 5.139, *p* < 0.05; main effect of orchiectomy) and FIN (50 mg/kg, IP) (F_1,36_ = 12.46, *p* < 0.001) significantly increased immobility; however, no interaction between these factors was detected (F_1,36_ = 2.16, NS), indicating that the depressogenic effects of FIN were not modified by the removal of gonads.

**Figure 8:**
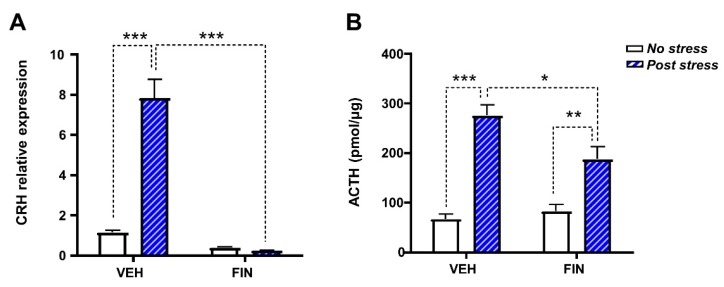
Finasteride (FIN, 50 mg/kg, IP) suppressed hypothalamus–pituitary–adrenal (HPA) axis responsiveness. (**A**) FIN completely suppressed the levels of transcript of corticotropin releasing hormone (CRH) in the paraventricular nucleus (PVN) of the hypothalamus, in rats subjected to forced-swim stress or non-stressful conditions (*n* = 4/group). (**B**) FIN also reduced plasma ACTH levels in stressed rats (*n* = 9–10/group). * *p* < 0.05, ** *p* 0.01, *** *p* < 0.001 for all comparisons indicated by dotted lines.

To further understand the mechanisms underlying the effects of FIN (50 mg/kg, IP) on the stress response, we tested the impact of this drug on CRH transcript levels in the PVN, as well as ACTH plasma concentrations at 30 min after FST. Two-way ANOVA analyses showed that, in line with previous results [36], CRH mRNA was increased by stress exposure (main effect of stress: F_1,10_ = 112.8, *p* < 0.001) and decreased by FIN (main effect of treatment: F_1,10_ = 69.51, *p* < 0.001). Furthermore, a significant treatment × stress interaction was found (F_1,10_ = 75.85, *p* < 0.001). *Post-hoc* comparisons revealed that FIN drastically reduced CRH mRNA levels in both stressed and unstressed rats (*p*’s < 0.001; Figure 8A). Furthermore, no differences in CRH transcript were found between FIN-treated rats, irrespective of their exposure to FST. The analysis of ACTH plasma levels showed a significant main effect of FIN (F_1,34_ = 70.68, *p* < 0.001) and FIN × stress interaction (F_1,34_ = 7.56, *p* < 0.01). *Post-hoc* comparisons showed that, as previously described, FST stress increased ACTH plasma levels [37] in both FIN- and vehicle-treated rats (VEH/no stress vs. VEH/stress, *p* < 0.001; FIN 50/no stress vs. FIN 50/stress, *p* < 0.01); however, FIN blunted the ACTH response in the stressed rats (VEH/stress vs. FIN 50/stress, *p* < 0.05; Figure 8B).

## 4. Discussion

The results of this study showed that, in Long–Evans rats, doses of FIN that did not intrinsically reduce locomotor activity attenuated the behavioral responses to a wide array of environmental stimuli, ranging from incentive and rewarding to stressful and aversive. The effects of FIN were first tested in a battery of conflict-based paradigms, including defensive withdrawal, novelty-induced hypophagia, and social interaction. These tests were aimed at measuring complementary facets of anxiety-like behavior as a function of the contrast between the rats’ innate avoidance of potentially threatening cues (including brightly lit or novel contexts and unfamiliar rats) and their propensity to engage in diversive exploration or consume palatable food. Under these experimental settings, FIN reduced the proclivity to transition from a brightly lit arena to a protected chamber, attenuated the motivation to eat cheese puffs in a novel cage, and decreased the overall duration of social investigation of foreign rats. To verify whether these responses reflected an actual enhancement of anxiety, the effects of FIN were re-tested in alternative versions of the same testing procedures, specifically designed to assess how behavioral reactivity could be modified by abating the ethological conflict between approach and avoidance. Our results showed that the avoidance-enhancing properties of FIN were not substantially modified by these conditions, suggesting that the primary effect of FIN was based on the reduction of goal-driven behaviors and arousal associated with salient stimuli, irrespective of the anxiogenic properties of the context and cues. In keeping with this interpretation, FIN reduced saccharin preference, a well-characterized index to measure reward sensitivity [38], but did not affect self-grooming, a behavior associated with anxiety and psychological burden secondary to stress [39]. In addition, FIN suppressed sensation-seeking and risk-taking behaviors in the delay discounting and wire-beam bridge paradigms. These results were reminiscent of preliminary clinical evidence pointing to a potential therapeutic effect of FIN in neuropsychiatric disorders featuring high impulsivity, such as problem gambling [25] and Tourette’s syndrome [24,40].

We also found that FIN increased the duration of the FST immobility, a behavioral parameter that measures of stress coping abilities in rodents [41]. Together with the reduction in saccharin or sucrose preference, this index has been long regarded as one of the most robust predictors of depression-like responses in animal models [42]. From this perspective, our findings were consistent with clinical reports indicating the association of FIN with depressive symptoms, including behavioral apathy and anhedonia [43]. Our experiments revealed that FIN yielded similar effects on stress reactivity in both sexes as well as in castrated males. Although FIN is only approved for clinical use in male patients, FIN is often used in women as a treatment for hirsutism, hair loss, and other hyperandrogenic conditions [44,45,46,47]. Thus, caution should be exercised in recommending FIN therapy in women with a well-known predisposition to depression and other psychological problems. Furthermore, our finding that FIN compounded the reduction of stress coping in orchiectomized male rats suggests that, in hypogonadic men, FIN might exacerbate their depressive symptoms, one of the most common manifestations observed in this patient group [48].

These findings also suggest that the depressogenic effects of FIN are likely not directly dependent on variations in peripheral sex hormones, but rather reflect the role of neural mechanisms. In agreement with this concept, we showed that FIN reversed the activation of the HPA axis (signified by the enhancement of CRH transcript and ACTH plasma levels) in response to acute FST stress. Several neurochemical processes may account for this effect. First, FIN blocks the synthesis of THDOC and other 5α-reduced neurosteroids, which promote CRH synthesis by activating GABAergic neurons in the PVN [49,50]. Second, FIN increases levels of progesterone, which has been described to exert negative effects on CRH synthesis in the PVN [51]. Third, by interfering with corticosterone degradation, FIN may enhance the levels of this hormone and potentiate its effects on the negative feedback regulation of CRH and ACTH synthesis [52,53].

Although our experiments did not directly assess a causal nexus between the variations in CRH and ACTH and FIN-associated behavioral changes, this link is in line with the key role of CRH in the promotion of physiological and behavioral responses to stress [54,55]. In particular, the idea that an acute reduction in CRH and ACTH levels may result in increased FST immobility has been indirectly supported by previous reports denoting the negative effects of these hormones on this behavioral response [56,57]. It is also worth noting that, in addition to its effects on stress response, CRH enables arousal and behavioral activation, irrespective of stress [58,59,60].

In addition to the reduction in HPA axis hormones, other mechanisms may be involved in the effects of FIN. For example, the effects of this drug on the reduction of responses to salient stimuli may reflect the marked antidopaminergic effects of FIN in the prefrontal cortex and nucleus accumbens, which have been documented by our previous studies [26,61,62,63]; indeed, dopaminergic signaling in these brain areas is pivotal to allowing for behavioral arousal and salience appraisal [64,65]. Future studies are needed to understand the involvement of the HPA axis, dopamine pathways, or other neural mechanisms in the behavioral effects of FIN.

In contrast with our findings, a recent report showed that subchronic, but not acute FIN increased forced-swim immobility in Wistar rats [66]. A possible explanation for these discrepancies might depend on specific variations in testing protocols or genetic background of rats, given the different effects of FIN across different rat strains [62]. Several limitations of our study should be acknowledged. First, our experiments did not qualify the specific 5αR isoform responsible for the effects of FIN. In humans, FIN has been shown to act as a potent inhibitor of 5αR type 2 (5αR2; IC_50_: 69nM), while it inhibits type 1 (5αR1) less effectively (IC_50_: 360 nM) [67]. However, in rats, FIN acts as a potent inhibitor of both 5αR1 and 5αR2 due to a tetrapeptide sequence encoded by the exon 1 of the rat gene that confers sensitivity to 5αR1 [68]. Although these two isoenzymes serve similar catalytic functions, they differ by intracellular and anatomical distribution [69,70,71]. In the CNS, while 5αR1 immunoreactivity is present in both neurons and glia, the distribution of 5αR2 is limited to some neurons (such as the pyramidal cells of the cortex) [69,72]. Thus, it is possible that some of our findings may not be fully translatable into complications observed in humans. Irrespective of these issues, the possibility that 5αR inactivation may be associated with depression-related outcomes is consistent with our previous studies documenting that chronic psychosocial stress leads to the downregulation of both isoforms [73].

Second, most effects reported in this study were limited only to the acute effects of FIN. Nevertheless, the increase in FST immobility reported in this study was similar to the effects of other regimen of FIN administration, including sub-chronic administration and after prolonged discontinuation of the drug [66,74]. Further investigations are warranted to verify whether the impairments in stress caused by these different regimens are underpinned by similar neurobiological processes.

Finally, our studies did not qualify which changes in steroid profiles are responsible for the behavioral effects of FIN. It is likely that changes in neurosteroids may be primarily responsible for these effects; indeed, several clinical trials have shown that AP levels are reduced in the CSF and plasma of depressed individuals [75] and animal models of chronic stress [76]. Furthermore, AP exerts anxiolytic and antidepressant properties in animal models and humans [77], and has been recently approved by FDA for the treatment of post-partum depression [78]. In addition to AP, however, other 3α,5α-reduced neuroactive steroids might participate in the behavioral complications induced by FIN, including the testosterone metabolites DHT and 3α-diol, which have been shown to exert positive effects on stress coping and motivation [8,9,79,80].

These limitations notwithstanding, the results of this study qualified the impact of FIN across a broad range of behavioral domains. Given the emerging evidence on the neuropsychiatric complications of FIN and its therapeutic potential in Tourette’s syndrome and pathological gambling, our findings may be critical for the understanding of the neurobehavioral mechanisms underpinning these outcomes and the development of novel steroid-based treatments that may preserve the therapeutic effects of FIN while reducing its liability for adverse events.
